# Supplementary material for: β-Cyclodextrin Nanophotosensitizers for Redox-Sensitive Delivery of Chlorin e6
Source: Molecules. 2023 Nov 2;28(21):7398. doi: 10.3390/molecules28217398 (PMC10648776; doi:10.3390/molecules28217398)
Supplement: Supplementary file 1 [file molecules-28-07398-s001.zip › molecules-2681681-supplementary.docx]

**Supplementary material**


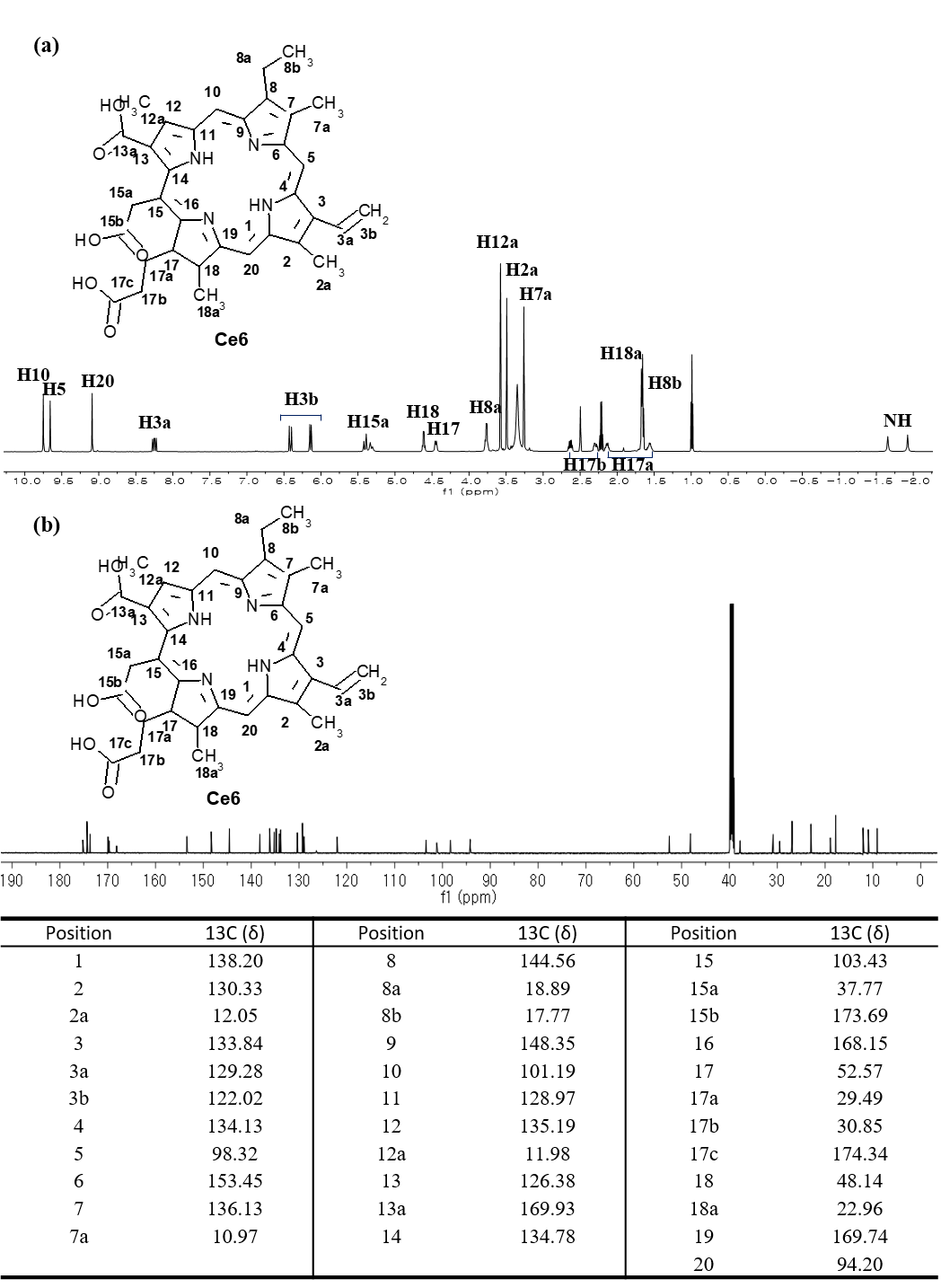


**Figure S1.** (a) ^1^H and (b) ^13^C NMR spectra of Ce6 in DMSO-d_6_.


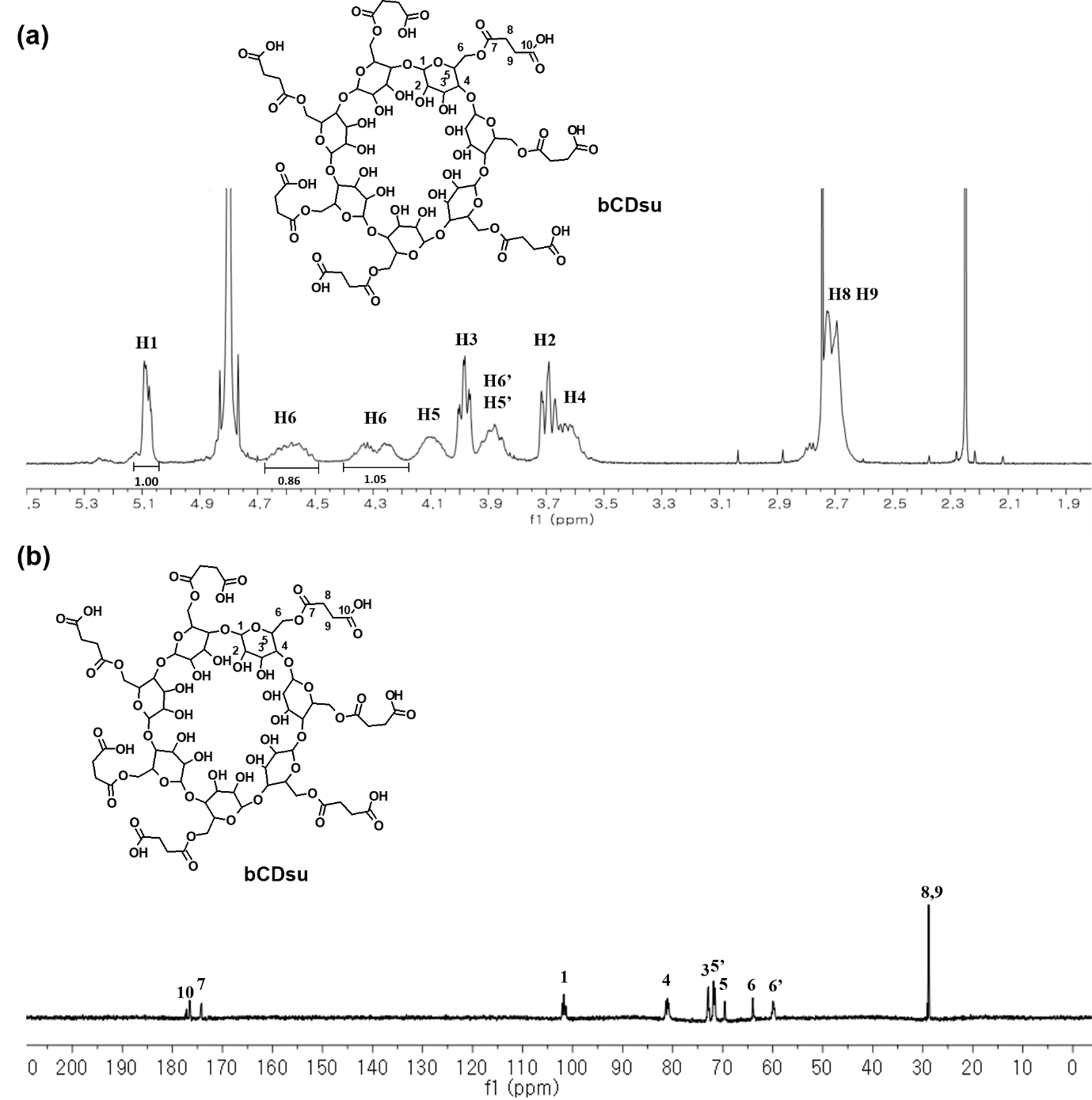


**Figure S2.** (a) ^1^H and (b) ^13^C NMR spectra of bCDsu in D_2_O,.

**
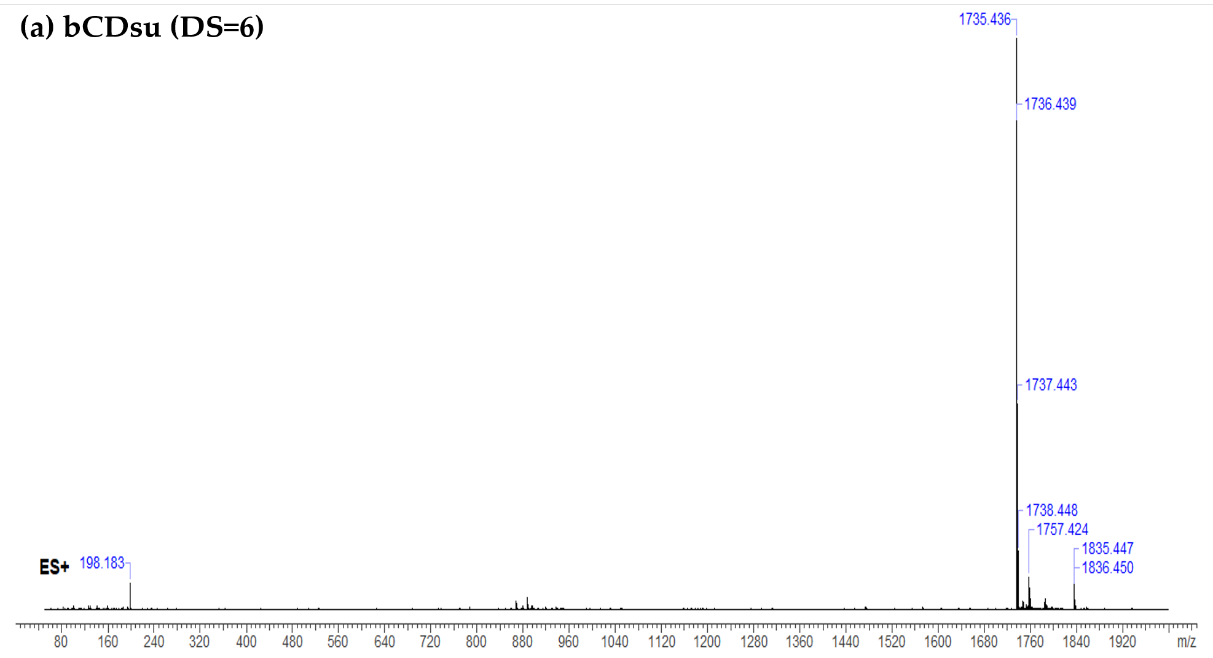
**

**
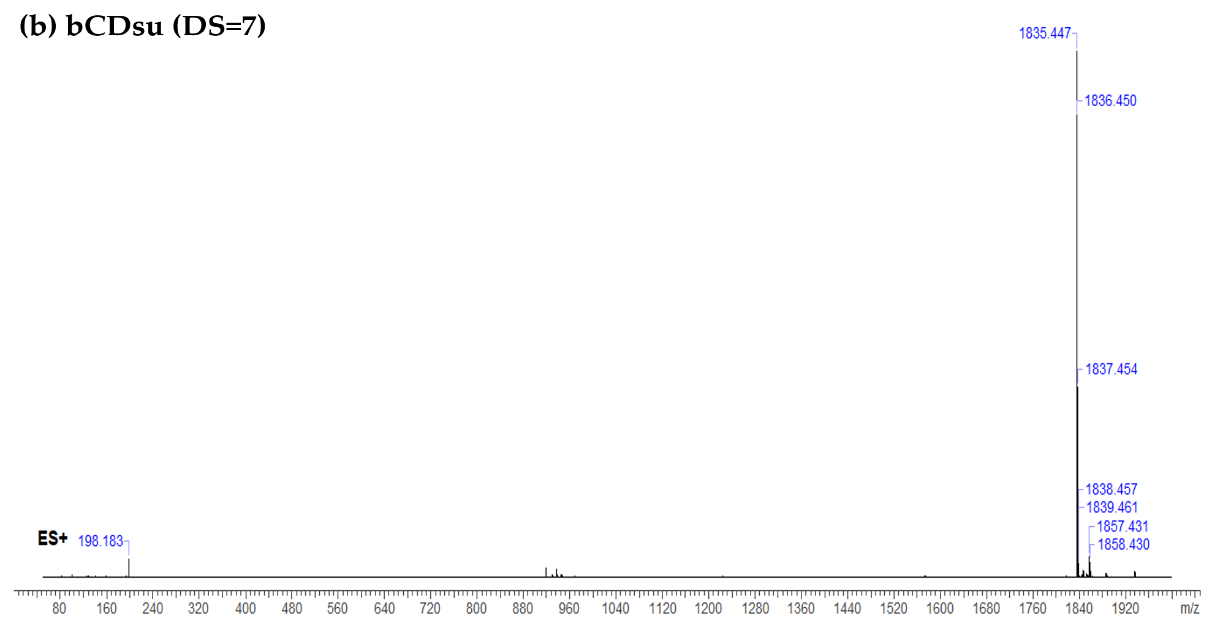

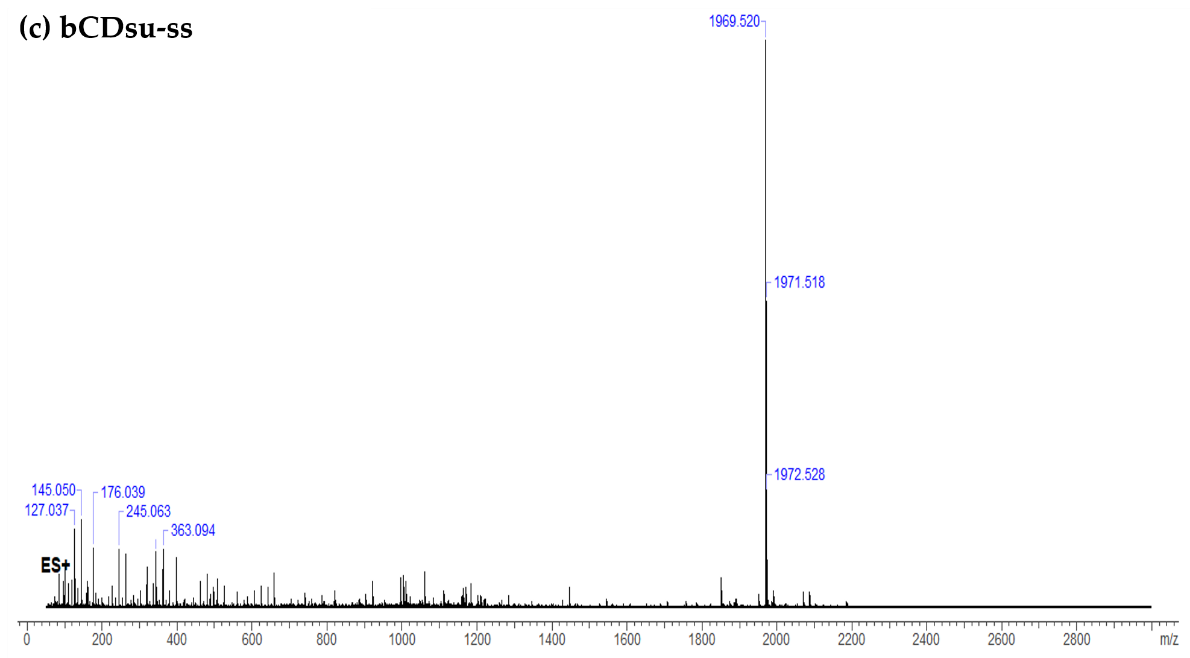
**

**Figure S3.** Mass spectra (MS) of (a) bCDsu (DS of succinyl group = 6), (b) bCDsu (DS of succinyl group = 7) and (c) bCDsu-ss.


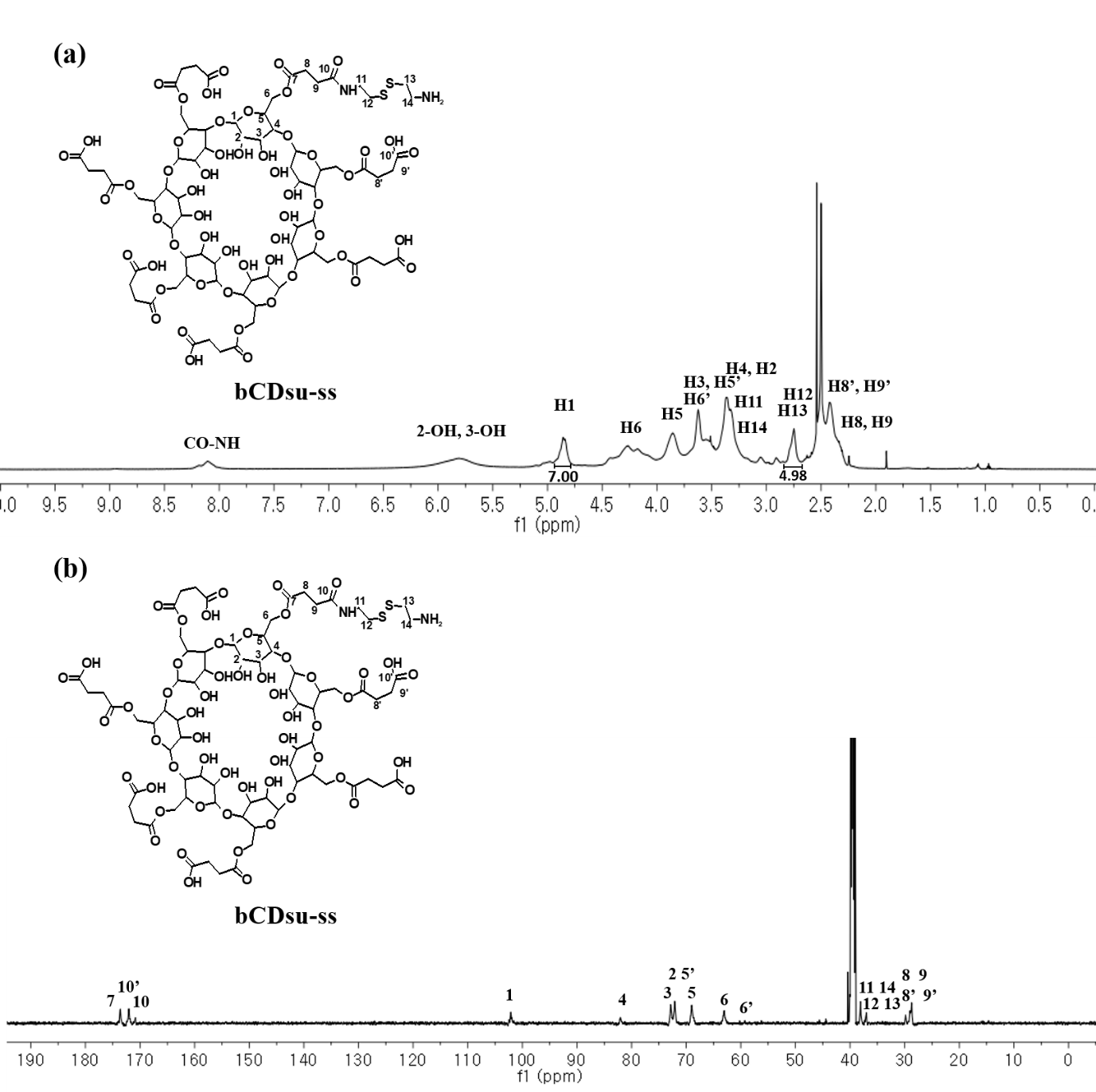


**Figure S4.** (a) ^1^H and (b) ^13^C NMR spectra of bCDsu-ss in DMSO-d_6_.


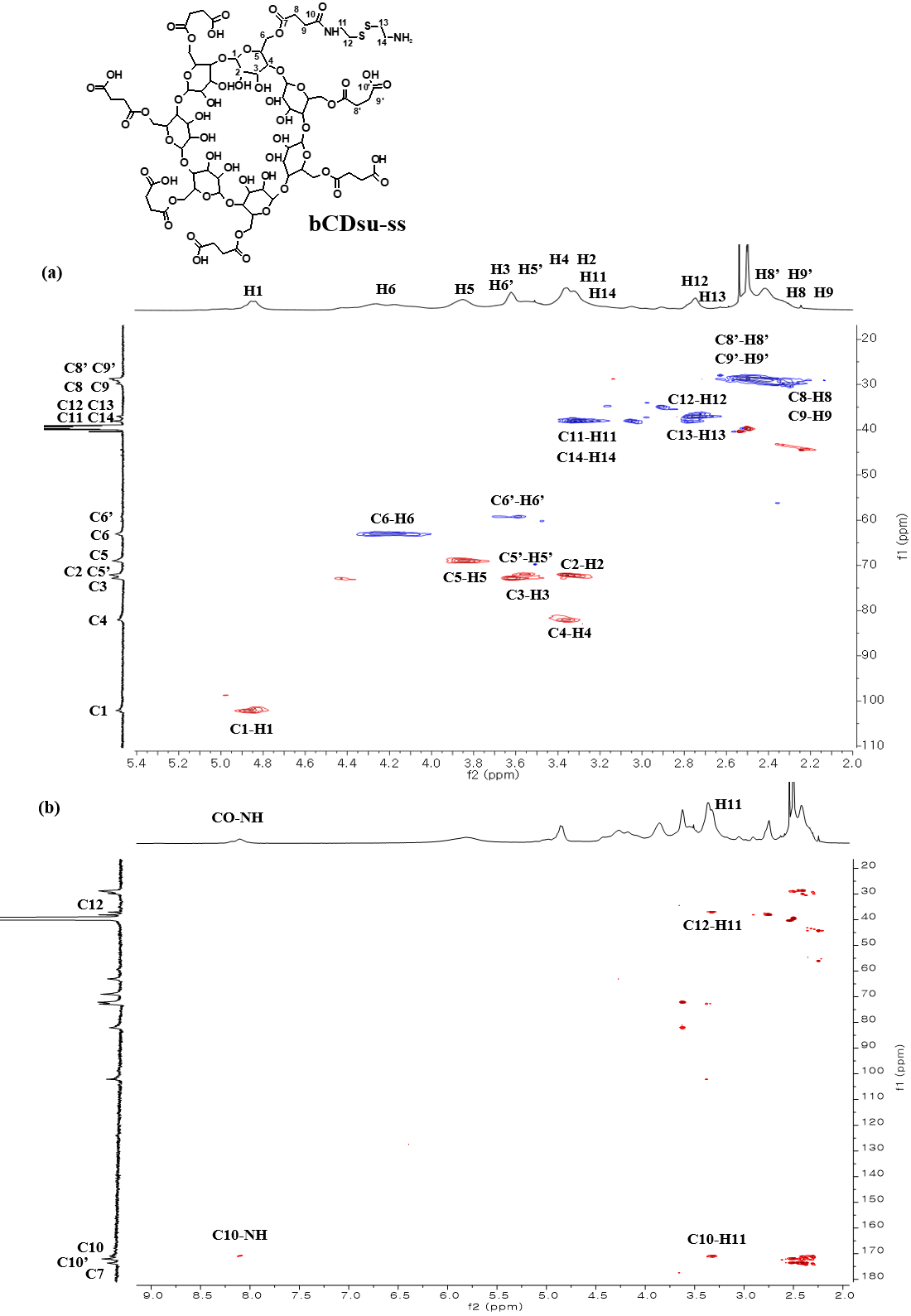


**Figure S5.** (a) HSQC and (b) HMBC of bCDsu-ss in DMSO-d_6_.


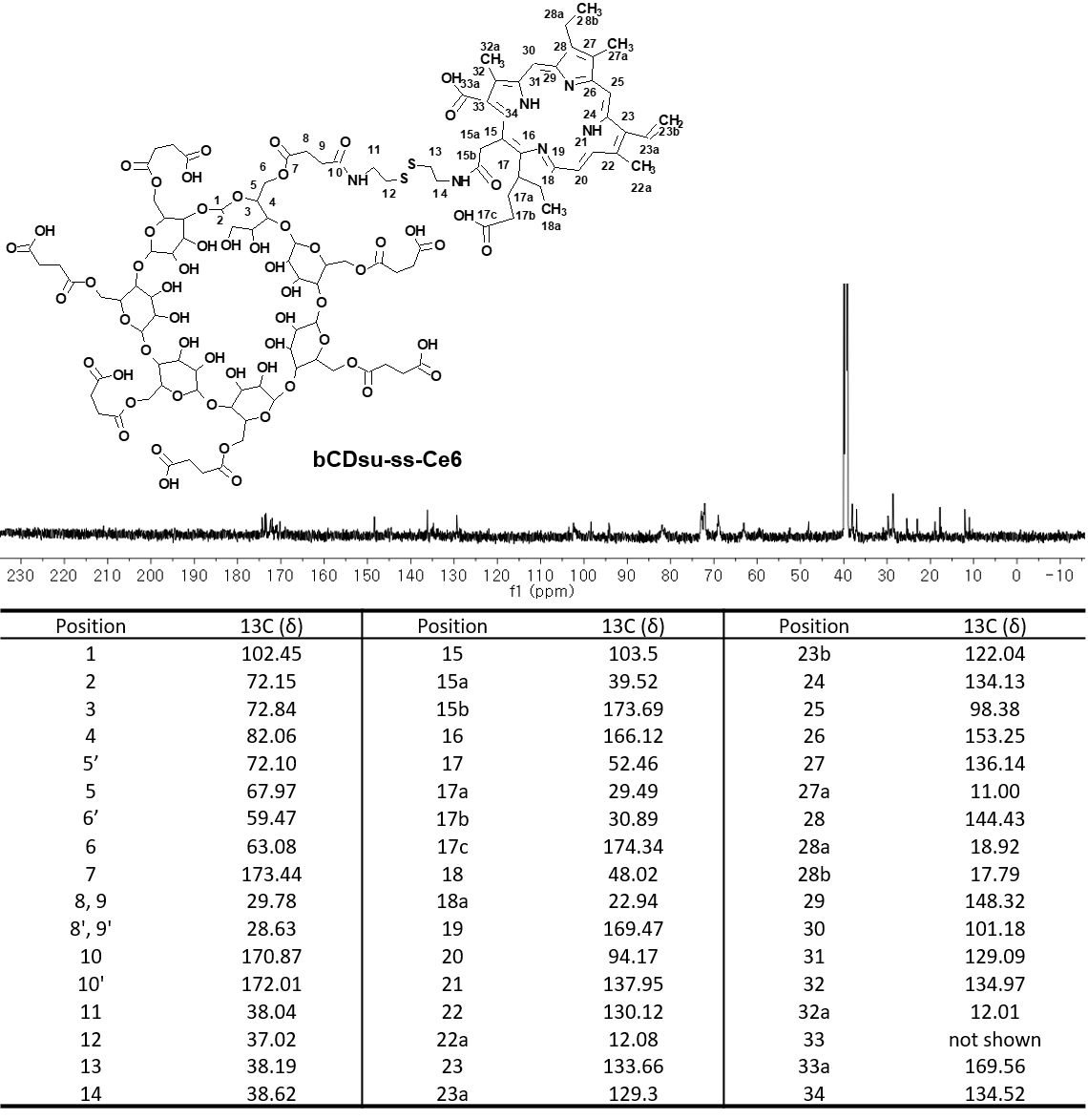


**Figure S6.** ^13^C NMR spectra of bCDsu-ss-Ce6 conjugates

**
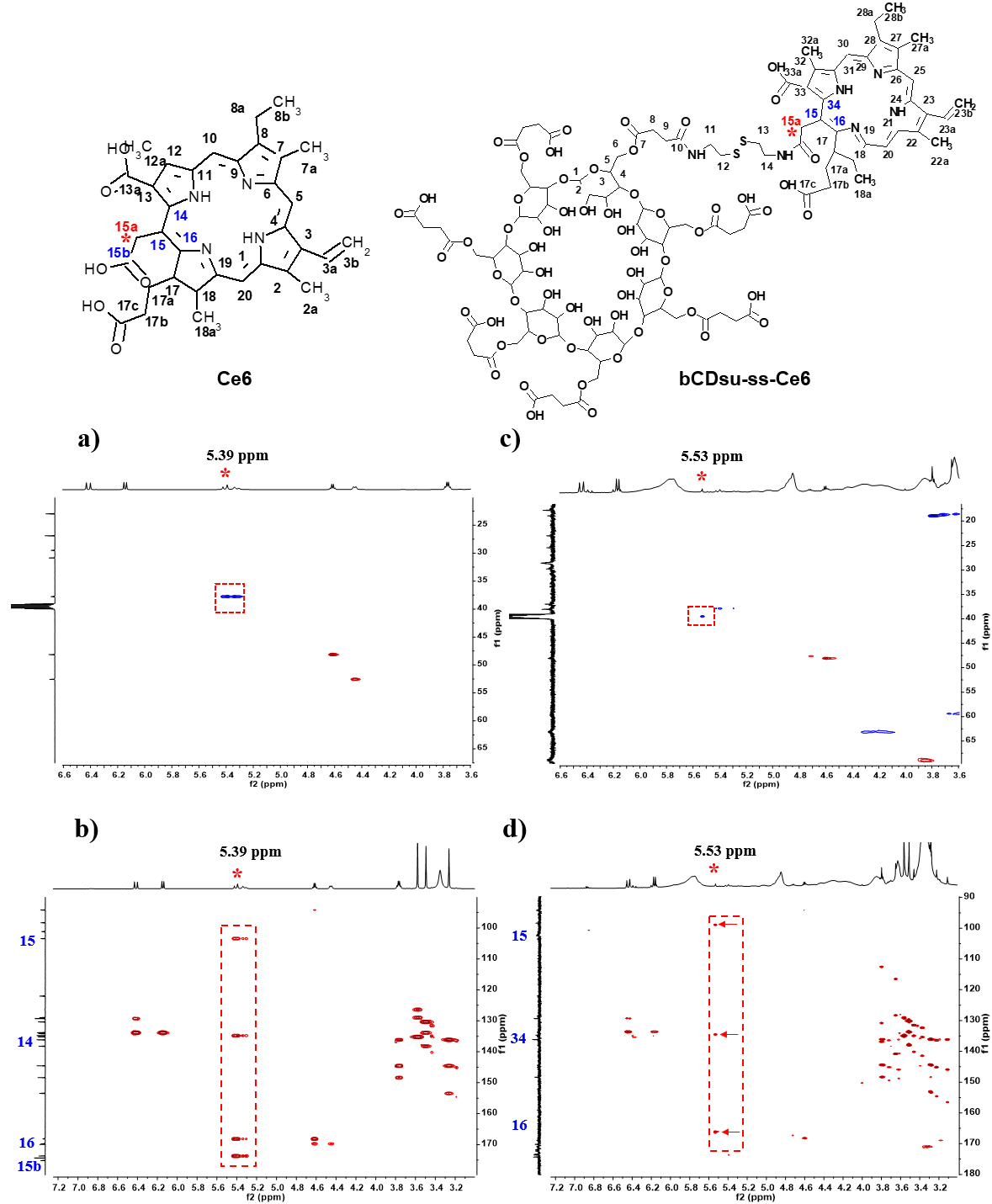
**

**Figure S7.** HSQC and HMBC of Ce6 and bCDsu-ss-Ce6 conjugates. HSQC (a) and HMBC (b) of Ce6; HSQC (c) and HMBC (d) of bCDsu-ss-Ce6 conjugates.


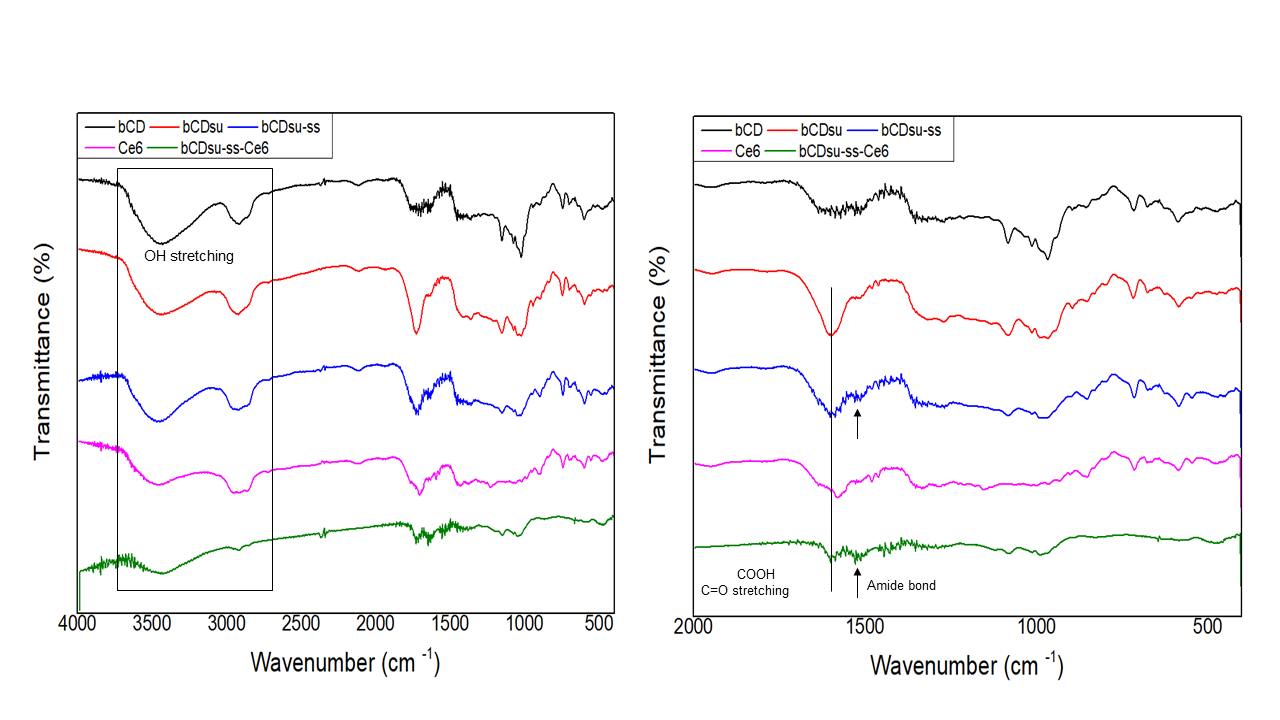


**Figure S8.** FT-IR spectra of bCD, bCDsu, bCDsu-ss, Ce6 and bCDsu-ss-Ce6 conjugates**.**
